# Differential Expression of ARG1 and MRC2 in Retinal Müller Glial Cells During Autoimmune Uveitis

**DOI:** 10.3390/biom15020288

**Published:** 2025-02-14

**Authors:** Amelie B. Fleischer, Barbara Amann, Christine von Toerne, Roxane L. Degroote, Adrian Schmalen, Tanja Weißer, Stefanie M. Hauck, Cornelia A. Deeg

**Affiliations:** 1 Chair of Physiology, Department of Veterinary Sciences, LMU Munich, D-82152 Martinsried, Germanytanja.weisser@tiph.vetmed.uni-muenchen.de (T.W.); 2 Metabolomics and Proteomics Core, Helmholtz Center Munich, German Research Center for Environmental Health, D-80939 Munich, Germany

**Keywords:** Retinal Müller glial cells (RMG), retinal neuroinflammation, equine recurrent uveitis (ERU), autoimmune uveitis, major histocompatibility complex class II (MHC class II), Arginase 1 (ARG1), mannose receptor C-type 2 (MRC2), Thrombospondin 1 (THBS1), atypical antigen presenting cell (APC), ocular immune privilege

## Abstract

Retinal Müller glial cells (RMG) play a crucial role in retinal neuroinflammation, including autoimmune uveitis. Increasing evidence supports their function as active modulators of immune responses and potential atypical antigen-presenting cells (APCs). To further investigate this hypothesis, we conducted a differential proteome analysis of primary equine RMG from healthy controls and horses with equine recurrent uveitis (ERU), a spontaneous model of autoimmune uveitis. This analysis identified 310 proteins with differential abundance. Among these, the Major Histocompatibility Complex (MHC) class II and the enzyme Arginase 1 (ARG1) were significantly enriched in RMG from uveitis-affected horses, whereas Mannose Receptor C-type 2 (MRC2) and its interactor Thrombospondin 1 (THBS1) were more abundant in healthy RMG. The detection of MHC class II in equine RMG, consistent with previous studies, validates the robustness of our approach. Furthermore, the identification of ARG1 and MRC2, together with THBS1, provides new insights into the immunomodulatory and antigen-presenting properties of RMG. Immunohistochemical analyses confirmed the proteomic findings and revealed the spatial distribution of ARG1 and MRC2. ARG1 and MRC2 are thus markers for RMG in the neuroinflammatory or physiological milieu and highlight potential differences in the immune function of RMG, particularly in antigen presentation.

## 1. Introduction

Retinal Müller glial cells (RMGs), the resident macroglial cells of the retina, have gained increasing attention in ophthalmic research due to their involvement in a wide range of retinal diseases [1,2,3,4,5]. RMGs span the entire thickness of the retina, from the outer limiting membrane (OLM) to the inner limiting membrane (ILM), contributing to the blood–retinal barrier (BRB) [6], regulating retinal water, pH, and ion homeostasis [7,8,9], and participating in neurotransmitter recycling in the healthy retina [10,11]. During retinal inflammation, RMG transition to a gliotic state with dual characteristics: initially ensuring neuroprotection but later contributing to neural damage [12,13,14]. Given their unique structure, extending across the entire retina, RMGs serve as central mediators of communication, interacting with neighboring retinal cells and infiltrating immune cells during retinal homeostasis and inflammation [15]. Under inflammatory conditions, as demonstrated primarily in rodent in vitro-models and other species, RMGs adopt a multifaceted inflammatory phenotype. This phenotype is characterized by the secretion of pro-inflammatory cytokines [16,17,18], the generation of reactive oxygen species (ROSs) [19], the expression of toll- like receptors [20,21], the secretion of chemokines [22,23], and the active participation in the phagocytosis of dying retinal cells [24,25]. However, many aspects of the exact role of RMG in retinal inflammation remain to be elucidated.

Autoimmune uveitis is a sight-threatening autoimmune disease and a significant cause of visual impairment in humans [26,27]. The pathogenesis of autoimmune uveitis is driven by autoreactive CD4^+^ T cells from the periphery, which cross an impaired BRB, infiltrate the immune-privileged inner eye, and target retinal autoantigens, causing detrimental inflammation and destruction [28]. Equine recurrent uveitis (ERU), a condition affecting horses worldwide [29,30,31], represents the only spontaneous animal model that collectively displays the clinical and pathophysiological hallmarks of autoimmune uveitis in humans, such as its relapsing-remitting character and CD4^+^ T cell-driven autoimmune etiology [32,33,34]. Furthermore, the immune systems of horses and humans are relatively similar [35,36]. By contrast, murine models of autoimmune uveitis are mostly non-recurrent [37], making them unsuitable for examining the relapsing nature of autoimmune uveitis observed in humans. Patients with autoimmune uveitis remain at risk of vision loss due to the lack of targeted therapeutic interventions, as uveitis pathogenesis is multifactorial and not fully understood [27]. Consequently, new insights into ERU pathogenesis, particularly the identification of novel markers for retinal inflammation, are of high translational relevance.

While it is known that CD4^+^ T cells drive disease pathogenesis in humans [38], mice [39], rats [40], and horses [41,42,43], it remains unclear how these T cells are activated—not only in the periphery but also within the eye itself. In uveitis, CD4^+^ T cells specifically target retinal autoantigens [43,44], which remain stably expressed, even as retinal integrity is destroyed in advanced stages of the disease [45]. This stability might contribute to the progressive and relapsing nature of ERU [45]. The exact triggers for these recurrent inflammatory episodes remain unknown to date. Antigen presentation by antigen-presenting cells (APCs) within the eye, via Major Histocompatibility Complex (MHC) class II, is critical for initiating immune responses by infiltrating CD4^+^ T cells [46]. However, the APC responsible for initiating and sustaining the CD4^+^ T cell response and retinal immunity in autoimmune retinal inflammation has not yet been precisely defined [46]. Notably, microglia, the retina’s resident immune cells, can express MHC class II in inflammatory settings, including autoantigen-induced experimental autoimmune uveitis (EAU) in mice [47,48]. While microglia have been proposed to play a part in initiating the immune response in EAU, their presence is not essential to sustain and prolong retinal inflammation and the CD4^+^ T cell response [49]. Moreover, it remains uncertain whether resident retinal microglia are capable of antigen presentation during autoimmune uveitis [46,50]. This uncertainty has sparked growing interest in other retinal cells that may contribute to and sustain the inflammatory process, with RMGs emerging as potential atypical APCs.

Given the unique position of RMGs in the retina and their integration into the BRB, infiltrating immune cells are likely to come in contact with activated RMG [51]. Our research has shown that RMGs actively secrete Interferon γ (IFN-γ) in the course of ERU, a hallmark T helper (Th) 1 cell cytokine, thereby triggering retinal inflammation and influencing the retinal immune environment [16]. Interestingly, MHC class II expression during ERU was shown in RMG [41] and in human patients with subretinal fibrosis and uveitis syndrome [52]. More recent studies have demonstrated that primary stimulated RMGs can express hallmark proteins of antigen presentation and T cell co-stimulation in vitro [17,53]. In murine models of EAU, increased MHC class II abundance in RMGs supports the hypothesis that these cells may not only present antigens but also recruit leukocytes during retinal inflammation [50,51]. While these findings highlight the versatile functions of RMG under pro-inflammatory conditions and in murine models, their precise role in autoimmune uveitis remains poorly understood. To address this gap, the primary objective of this study was to investigate the protein expression profile of RMG in both healthy and diseased states and to define markers to distinguish between these two phenotypes. By distinguishing activated uveitic RMG from their healthy counterparts, we aimed to uncover the molecular mechanisms driving the breakdown of ocular immune privilege during the progression of autoimmune uveitis. Through differential proteome analysis, we provide new insights into the role of RMG in retinal neuroinflammation and disease pathogenesis.

## 2. Material and Methods

### 2.1. Retinal Specimen

For this study, we used a total of ten control and eleven ERU-diseased eyes. In particular, three healthy and three uveitic eyes were used for the preparation of primary RMG and differential proteome analysis (*n* = 3 biological replicates per group, with *n* = 1 technical replicate per sample). For immunohistochemical analysis, we used seven healthy control eyes and eight uveitic eyes obtained from our tissue biobank [7]. The immunohistochemical analysis was conducted with one technical replicate per sample. For the immunohistochemical analysis of Arginase 1 (ARG1) staining, we used six healthy and six diseased eyes (*n* = 6 biological replicates per group). For the immunohistochemical analysis of Mannose Receptor C-type 2 (MRC2) staining, we used five healthy and five diseased eyes (*n* = 5 biological replicates per group). Some of the samples were used for both immunohistochemical experiments, but not all. Healthy control eyes were collected from a local abattoir. The collection and use of equine eyes from the abattoir and cooperating equine clinics for the purpose of scientific research was approved by the corresponding board of the veterinary inspection office, Munich, Germany (permit number: DE-09-184-0063-21). Uveitic eyes were obtained from horses undergoing enucleation for therapeutic purposes in collaboration with local veterinary clinics. Clinical diagnoses of uveitis were made by experienced veterinary ophthalmologists based on a documented history of at least three relapsing-remitting inflammatory episodes and clinical symptoms consistent with uveitis [54]. All procedures adhered to ethical principles and guidelines for scientific experiments on animals, following the ARVO Statement for the Use of Animals in Ophthalmic and Vision Research. Importantly, no experimental animals were used in this study.

### 2.2. Differential Proteome Analysis

To prepare primary RMGs, eyes were processed immediately after enucleation. Residual tissue was removed, and the eyecups were disinfected with 80% ethanol. Under sterile conditions, eyeballs were opened circumferentially to expose the posterior chamber. Retinas were carefully separated from the vitreous and retinal pigment epithelium, mechanically disintegrated with micro scissors, and enzymatically digested with papain (Carl Roth, Karlsruhe, Germany) for 30 min at 37 °C. Papain was activated by incubation with 1.1 mM ethylenediaminetetraacetic acid (EDTA), 0.067 mM mercaptoethanol, and 5.5 mM cysteine HCl (all reagents: Merck, Darmstadt, Germany) for 40 min at 37 °C. The enzymatic reaction was stopped by adding Dulbecco’s Modified Eagle Medium (DMEM, Pan Biotech, Aidenbach, Germany), supplemented with 10% fetal bovine serum (FBS, Merck, Darmstadt, Germany). The cells were triturated after adding deoxyribonuclease I (Merck, Darmstadt, Germany) and then collected by centrifugation. After collection, the cells were resuspended in DMEM supplemented with 10% FBS and 1% penicillin-streptomycin (P/S, Pan Biotech, Aidenbach, Germany) and seeded into six-well plates (Sarstedt, Nümbrecht, Germany). Non-adherent cells were removed after 24 h, and the medium was replaced repeatedly to obtain pure RMG cultures, as previously described [55]. Once 80–100% confluency was reached, cells were split into 75 cm^2^ flasks using 1 mM trypsin EDTA (Thermo Fisher Scientific, Dreieich, Germany) and cultured at 5% CO_2_ and 37 °C. The second passage of each cultured flasks was used, and cells were cultured for two weeks. Supernatants were routinely tested for contamination with *Mycoplasma* spp. via PCR (Bio-Techne, Wiesbaden, Germany), yielding negative results. To remove residual FBS, cells were washed twice with serum-free DMEM containing 1% P/S, followed by a one-hour incubation in the same medium. Cells were then incubated overnight (~16 h) in serum-free DMEM with 1% P/S to prevent interference from FBS-derived cytokines in cell–cell communication. Following incubation, cells were washed with phosphate-buffered saline (PBS), lysed in PBS containing 1% Nonidet P40 (Roche, Grenzach-Wyhlen, Germany), and detached using a cell scraper. Lysates were transferred to low-binding tubes (Sarstedt, Nümbrecht, Germany), vortexed repeatedly, and intermittently incubated on ice.

### 2.3. Liquid Chromatography-Mass Spectrometry (LC-MS/MS) and Quantitative Analysis

Protein concentration was determined using Pierce BCA protein assay (Thermo Fisher Scientific, Dreieich, Germany). A total of 10 μg of each sample were digested with Lys-C and trypsin using a modified filter-aided sample preparation (FASP) procedure, as previously described [56,57]. Equal peptide amounts per sample were measured on a Q-Exactive HF-X mass spectrometer (Thermo Fisher Scientific, Waltham, MA, USA) coupled online to an Ultimate 3000 nano-RSLC (Thermo Fisher Scientific, Dionex, Waltham, MA, USA). Tryptic peptides were automatically loaded on a C18 trap column (300 µm inner diameter × 5 mm, Acclaim PepMap100 C18, 5 µm, 100 Å, Thermo Fisher Scientific, Waltham, MA, USA) prior to C18 reversed-phase chromatography on the analytical column (nanoEase MZ HSS T3 Column, 100 Å, 1.8 µm, 75 µm × 250 mm, Waters, Rydalmere, NSW, Australia) at 250 nL/min flow rate in a 95 min non-linear acetonitrile gradient from 3 to 40% in 0.1% formic acid. Profile precursor spectra from 300 to 1500 *m*/*z* were recorded at 60,000 resolution with an automatic gain control target of 3 × 10^6^ and a maximum injection time of 30 ms. Subsequently, TOP15 fragment spectra of charges 2 to 7 were recorded at 15,000 resolution with an AGC target of 1 × 10^5^, a maximum injection time of 50 ms, an isolation window of 1.6 *m*/*z*, normalized collision energy of 28, and a dynamic exclusion of 30 s.

### 2.4. Protein Identification, MS Label-Free Quantification

Peptide and protein identification were carried out using Proteome Discoverer 2.5 (Thermo Fisher Scientific, Waltham, MA, USA) via a Sequest HT database search against the Ensembl horse database (Release 75: 22491 sequences in Proteome Discoverer), including human gene name orthologues for equine genes as defined by the HUGO Gene Nomenclature Committee (HGNC). Full tryptic specificity was applied, allowing one missed cleavage. The precursor mass tolerance was set to 10 ppm, and the fragment mass tolerance was set to 0.02 Da. Carbamidomethylation of cysteine was defined as a static modification, while deamidation of asparagine and glutamine, methionine oxidation, and methionine loss with N-terminal acetylation were set as dynamic modifications.

Percolator validated peptide spectrum matches (PSMs) and peptides, accepting only the top-scoring hit for each spectrum with false discovery rates (FDR) < 1% and posterior error probability < 0.05. A Sequest HT Xcorr filter threshold of 1.6 was applied, restricting further analysis to high-confidence matches only. The final protein list adhered to the strict parsimony principle.

Quantification was based on the abundance values of the top three unique peptides, normalized against total abundance to account for sample loading errors. Ratios between experimental groups were calculated as medians of all sample and peptide comparisons. Statistical significance was determined using background-based *t*-tests as described [58], based on the presumption that expression changes are being examined for a limited number of proteins compared to the total number of proteins quantified. The quantification variability of the non-changing “background” proteins can be used to infer which proteins change their expression in a statistically significant manner. The calculated *p*-values were adjusted for multiple testing using the Benjamini–Hochberg correction [59], resulting in adjusted *p*-values (adj. *p*). Protein identifications supported by fewer than two unique peptides were excluded from the analysis.

### 2.5. Data Processing, Visualization, and Analysis

Where no human gene orthologue was deposited in the database, equine Accession IDs were used. Statistically significant proteins with an adj. *p* of ≤0.05 were considered differentially abundant with a two-fold abundance change (ERU/healthy ratio more abundant proteins of 2 or higher; ERU/healthy ratio less abundant proteins 0.5 or lower). Abundance ratios were capped at 100 or 0.01. Proteins exclusively quantified in one of the two groups received the maximal or minimal abundance ratio, depending on whether they were exclusively quantified in healthy controls or ERU samples, respectively. To simplify data presentation, reciprocal ratio values were used for proteins more abundant in the healthy state, resulting in abundance ratios of 2 or higher for Healthy/ERU comparisons. To visualize the proteomic data, a Volcano Plot was generated in R (version 4.3.1, R Core Team (2024); Vienna, Austria, https://www.R-project.org, accessed on 3 September 2024) with the ggplot2 package (version 3.5.1).

Pathway enrichment analysis of the differentially abundant proteins (adj. *p* of ≤0.05; ratio ERU/healthy or healthy ERU ≥ 2) was conducted with open-source software Reactome (v91, https://reactome.org/, accessed on 24 January 2025). Over-representation of pathways was determined with hypergeometric distribution corrected for false discovery rate (FDR)/adj. *p*-value using the Benjamini–Hochberg correction.

The proteins selected for further analysis were chosen based on their strong association with key biological processes relevant to this study. This includes roles in antigen presentation, immune modulation, and inflammatory pathways. Additionally, proteins with potential immunosuppressive functions and in tissue homeostasis were prioritized to represent the characteristics of healthy cellular states. Potential interactors of the selected proteins were also taken into consideration for better insights into the functional dynamics underlying ERU pathogenesis.

### 2.6. Verification of Protein Candidates ARG1 and MRC2 from Differential Proteome Analysis via Immunohistochemical Staining

Immunohistochemical staining was performed on paraffin-embedded posterior ocular segments. Eyecups were processed for immunohistochemistry as previously described [60]. Retinal tissue samples were sectioned into 8 μm slices and mounted on coated slides (Superfrost Plus, R. Langenbrinck, Emmendingen, Germany). Heat antigen retrieval was conducted at 99 °C with 0.1 M EDTA-NaOH buffer (pH 8.0) for 15 min, followed by citrate-buffer (pH 6.6) (Merck, Darmstadt, Germany) for an additional 15 min. To minimize non-specific antibody binding, retinal sections were blocked with Tris-buffered saline containing Tween 20 (TBS-T; Tween 20, Merck, Darmstadt, Germany) supplemented with 1% bovine serum albumin (BSA, Serva, Heidelberg, Germany) and 5% goat serum, matching the host species of the secondary antibody. Specific protein detection was performed using specific primary antibodies, as follows. To visualize RMG morphology, monoclonal mouse anti horse-Vimentin (dilution 1:400, Merck, Darmstadt, Germany) was used, followed by staining with a goat anti mouse IgG H+L conjugated to Alexa Fluor 488 (dilution 1:500, Invitrogen, Dreieich, Germany). To detect Arginase 1 (ARG1) expression, we used polyclonal rabbit anti-human ARG1 (dilution 1:200, with the antibody targeting an epitope that is 100% homologous to the equine epitope, as stated by the manufacturer, Lifespan Biosciences, Eching, Germany). To assess MRC2 expression, polyclonal rabbit anti-human Mannose Receptor C-type 2 (MRC2) (dilution 1:50, Novus Biologicals, Wiesbaden, Germany) was applied. Sequence homology of the antibody binding site (stated by the manufacturer) to equine MRC2 was confirmed using NCBI’s Basic Local Assignment Search Tool (BLAST, https://blast.ncbi.nlm.nih.gov/Blast.cgi, accessed on 21 May 2024). To visualize ARG1 and MRC2 staining, a goat anti-rabbit IgG H + L coupled to Alexa Fluor 568 (dilution 1:500, Invitrogen, Dreieich, Germany) was used. Cell nuclei were counterstained with 4′,6-diamidino-2-phenylindole (DAPI, dilution 1:1000 Thermo Fisher Scientific, Dreieich, Germany). Fluorescent mounting medium (Serva, Heidelberg, Germany) was used to mount the retinal sections with glass coverslips.

### 2.7. Quantification Protein Candidate Expression in Equine RMG

Fluorescent images were acquired with the Leica DMi8 microscope (Leica Microsystems, Wetzlar, Germany) and quantified with Leica Application Suite X software, version 3.7.4.34563 (Leica Microsystems, Wetzlar, Germany). Protein expression of ARG1 and MRC2 was quantified as mean fluorescence intensity (MFI) in RMG of healthy controls and ERU-cases. To ensure accurate quantification of spatial expression in RMGs, regions of interest (ROIs) were manually selected to encompass entire RMGs. The localization of RMGs was determined by combining differential interference contrast (DIC) imaging with Vimentin staining.

The factor of MFI values was used to statistically analyze the differences in fluorescence intensity between healthy controls and ERU cases. Gaussian distribution was determined with the Kolmogorov–Smirnov test. As the data were normally distributed, Student’s *t* test was employed for statistical analysis. In case of significantly differing variances, the Welch correction was applied to account for these differences accurately. Statistical significance was set at *p* ≤ 0.05, with an asterisk indicating the level of significance (0.01 < * *p* ≤ 0.05). Data processing, analysis, and visualization were performed using GraphPad Prism version 5.04. Data are presented as mean +/− standard deviation (SD).

## 3. Results

### 3.1. Proteomes of RMG Differed Significantly in Healthy and Uveitic State

**Figure 1:**
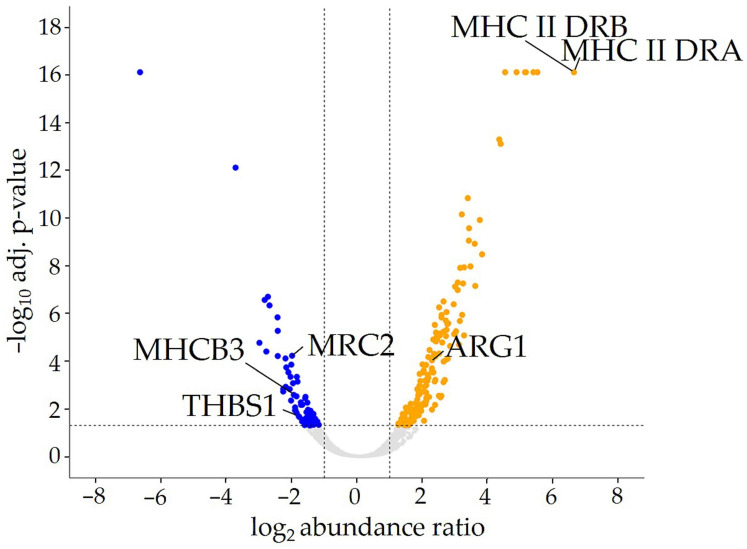
Volcano plot illustrating the differential abundance of proteins between control retinal Müller glial cells (RMG) and RMG from equine recurrent uveitis (ERU) cases. Among the identified proteins, 99 that are significantly more abundant in healthy RMG are marked in blue, while 211 that are more abundant in uveitic RMG are marked in orange. The dotted gray lines indicate thresholds for statistical significance (adj. *p* ≤ 0.05) and abundance ratio changes (ERU/healthy ≥ 2 or ERU/healthy ≤ 0.5). Proteins of particular interest are labeled with their corresponding HUGO Gene Nomenclature Committee (HGNC) gene symbols.

Differential proteome analysis of primary RMG from healthy and diseased horses revealed a total of 4198 identified and quantified proteins (Figure 1). Among these, 310 proteins exhibited differential abundance between healthy controls and ERU cases (Figure 1; Table S1). Specifically, 211 proteins were significantly more abundant (≥2.0-fold; adj. *p* ≤ 0.05) in the uveitic state, while 99 proteins were significantly more abundant (≥2.0-fold; adj. *p* ≤ 0.05) in the healthy state (Figure 1; Table S1). Since we were interested in finding novel markers for uveitic and healthy RMG and to further validate our hypothesis of RMG as atypical APC, we selected proteins with associations to processes in antigen presentation, inflammation, and immunomodulation for further analysis (Figure 1).

Among the proteins identified exclusively in RMG from ERU horses were two members of the MHC class II complex family: the MHC class II DR alpha chain (MHC II DRA) and beta chain (MHC II DRB) (Figure 1). These hallmark proteins of APCs were significantly more abundant in the proteome of uveitic RMG (adj. *p* ≤ 0.0001) (Figure 1) and were exclusively quantified in diseased RMG (Table 1). Interestingly, ARG1 was also significantly more abundant in the proteome of uveitic RMG compared to healthy controls (adj. *p* = 0.0001) in the proteome of diseased RMG compared to healthy controls (Figure 1), with a 4.9-fold increase in abundance (Table 1). ARG1 is a cytosolic enzyme commonly recognized as a classical polarization marker for human and murine macrophages in vitro, which are professional APCs [61,62]. To date, its role in ERU has not yet been explored.

To investigate how RMG function changes during the progression of ERU and the role of RMG in maintaining the immune privilege of the inner eye, we also examined proteins that were more abundant in healthy RMG and correspondingly lower in uveitic RMG (Table 2). Among these, MHC class I heavy chain (MHCB3) was significantly more abundant in the proteome of healthy RMG (adj. *p =* 0.0026) (Figure 1). MHCB3 abundance is 3.8-fold higher in healthy RMG compared to uveitic RMG (Table 1). MHC class I is expressed by all nucleated cells and is essential in presenting endogenous antigenic peptides on the cell surface [63]. Additionally, it is involved in the cross-presentation of exogenous peptides [63]. Moreover, we found the matricellular protein Thrombospondin 1 (THBS1) to be significantly more abundant in healthy RMG (adj. *p* = 0.0148) in the proteome of healthy RMG compared to uveitic RMG (Figure 1). The abundance of THBS1 was 3.6-fold higher in healthy RMG compared to RMG from uveitis cases (Table 2). Additionally, the transmembrane c-type-lectin- and collagen-receptor MRC2 was significantly more abundant in healthy RMG (adj. *p* = 0.0001; Figure 1), with a 4-fold higher abundance in healthy RMG compared to uveitic RMG (Table 2). Notably, MRC2 has not yet been described in the retina, in RMG or in the context of uveitis. Interestingly, THBS1 has been described as an interactor of MRC2, suggesting a potential functional link between these proteins [64].

Pathway enrichment analysis was performed on proteins that were differentially abundant in both the diseased and the healthy states. Overrepresented pathways in the diseased state included “Interferon signaling”, “Interferon alpha/beta signaling”, “Interferon gamma signaling” and “Oxidative Stress Induced Senescence”. The significantly enriched pathways (adj. *p* ≤ 0.05) are listed in Table S2.

Among the proteins that were not differentially abundant but were still constitutively expressed in RMG, were several costimulatory factors and adhesion molecules, including CD40, intracellular adhesion molecule 1 (ICAM1), CD81, CD9, CD48, and CD58 (Figure S1). Furthermore, lysosome-associated membrane proteins LAMP1 and LAMP2 were also constitutively expressed by RMG although not differentially abundant (Figure S1).

### 3.2. Spatial Distribution and Expression of ARG1 in Equine RMG with Significantly Higher Expression in Uveitic RMG

**Figure 2:**
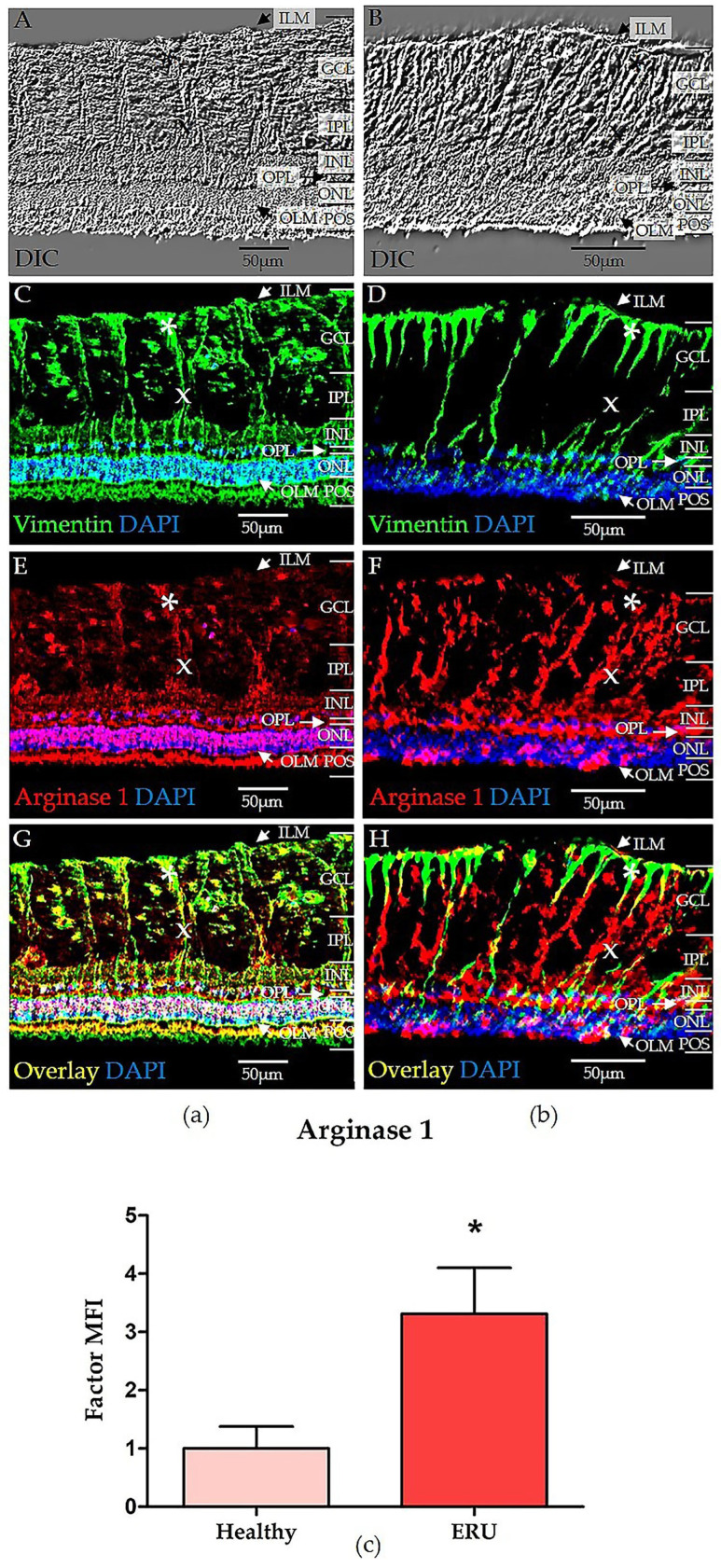
Equine RMG differentially express Arginase 1 (ARG1) in healthy (panel (**a**)) and uveitic states (panel (**b**)). ARG1 expression in RMG was quantified as mean fluorescence intensity of immunohistochemical staining (panel (**c**)). Retinal structure was visualized using differential interference contrast (DIC) microscopy in both healthy (**A**) and diseased (**B**) retinas. Vimentin staining (green) highlighted RMG structure and morphology in the healthy (**C**) and diseased (**D**) retinas. In diseased retinas, RMG appeared more prominent, indicating the onset of inflammation and exhibiting mild gliosis (**D**). ARG1 expression (red) was observed in both healthy (**E**) and diseased (**F**) retinas, with markedly stronger expression in the diseased retina (**F**). In healthy retinas, ARG1 was evenly distributed from the inner nuclear layer (INL) to the outer limiting membrane (OLM) (**E**). In uveitic retinas, ARG1 expression extended beyond the RMG, encompassing parts of the INL, outer plexiform layer (OPL), and ganglion cell layer (GCL) (**F**). In healthy RMG, ARG1 was moderately expressed in the endfeet (marked with *), perikarya located in the INL, and distal processes in the outer nuclear layer (ONL), while expression was weaker in the inner stem processes (marked with x). In uveitic RMG, ARG1 expression was stronger in the inner stem processes (x) and perikarya in the INL, while it decreased in the endfeet (*) and distal processes in the ONL. The ONL signal in uveitic RMG displayed a spotted pattern (**F**), distinct from the even distribution seen in healthy RMG (**E**). Overlay images of Vimentin (green) and ARG1 (red) confirmed the colocalization of ARG1 with RMG in both healthy (**G**) and uveitic (**H**) retinas. Images were captured at 630× magnification, and cell nuclei were counterstained with 4′,6-diamidino-2-phenylindole (DAPI). Representative images were selected from biological replicates that best displayed the observed expression patterns. ARG1 expression was increased 3.3-fold in uveitic RMG (panel (**c**), red bar, right) compared to healthy controls (panel (**c**), pink bar, left). The difference in expression was statistically significant (* *p* = 0.0351), with the asterisk indicating a level of significance with 0.01 < * *p* ≤ 0.05. The bar plot shows the mean fluorescence intensity (MFI) value ± standard deviation (SD), based on quantification of MFI in RMG from both control and uveitis cases. Retinal layers are listed from the innermost to the outermost layer as follows: inner limiting membrane (ILM); ganglion cell layer (GCL); inner plexiform layer (IPL); inner nuclear layer (INL); outer plexiform layer (OPL); outer nuclear layer (ONL); outer limiting membrane (OLM); photoreceptor outer segments (POS).

Next, we investigated the expression and precise distribution of ARG1 in healthy retinas (Figure 2A, DIC) and retinas from uveitis cases (Figure 2B, DIC). The RMG marker Vimentin was used to show RMG localization and morphology (Figure 2C,D). In this study, our goal was to investigate the early stages of retinal inflammation. In healthy retinas, RMG exhibited a characteristic columnar shape, extending from the ILM to the OLM (Figure 2C). In diseased retinas, RMG appeared more prominent, consistent with the early stages of inflammation, displaying features of a mildly gliotic phenotype (Figure 2D). RMG perikarya are located in the inner nuclear layer (INL) of the retina [9]. The inner stem process (Figure 2, marked with x) and outer stem process extend in opposite directions from the perikaryon [10]. The inner stem process terminates in a funnel-shaped endfoot (Figure 2, marked with *) in the ganglion cell layer (GCL), adjacent to the ILM [10]. In the outer nuclear layer (ONL), the outer stem process branches into distal processes that envelop the perikarya of photoreceptors [9].

ARG1 expression was detected in both healthy (Figure 2E) and diseased retinas (Figure 2F), with prominent expression in all RMG. Other retinal layers also stained positively for ARG1. In healthy retinas, ARG1 expression outside of RMG was distributed across the distal segment, from the OLM, where it formed a distinct, thin band of expression, to the INL (Figure 2E). Notably, the ONL was markedly positive for ARG1 (Figure 2E). In contrast, uveitic retinas exhibited markedly higher ARG1 expression with a clear shift in both intensity and spatial distribution (Figure 2F). ARG1 in uveitic retinas extended beyond RMG to encompass the outer plexiform layer (OPL), the INL, and parts of the GCL. Notably, the ONL in uveitic retinas displayed a spotted and irregular ARG1 expression pattern (Figure 2F), deviating from the even distribution seen in the healthy ONL (Figure 2E). Furthermore, ARG1 expression was absent in the OLM in uveitic retinas (Figure 2F).

**Figure 3:**
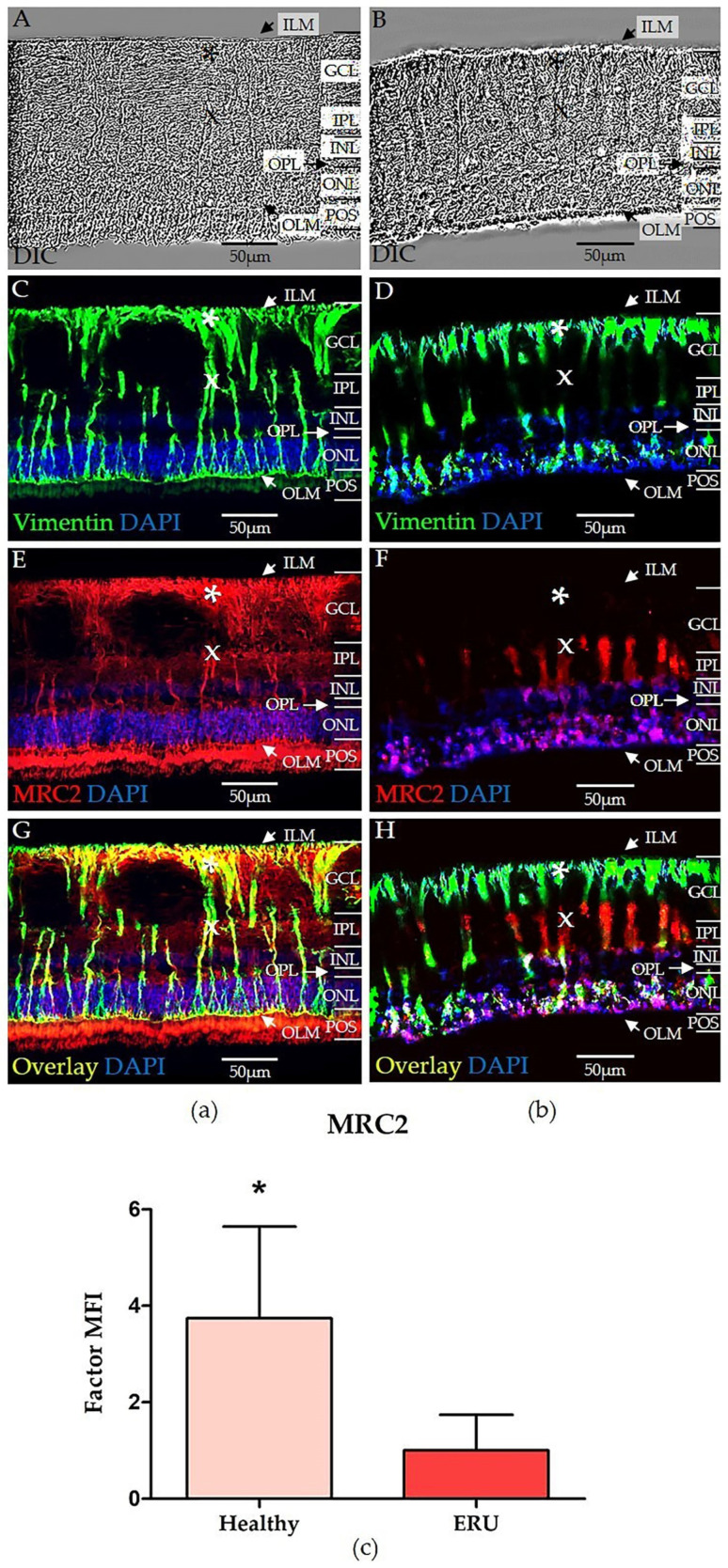
Equine RMG differentially express Mannose Receptor C-type 2 (MRC2) in both healthy (panel (**a**)) and uveitic (panel (**b**)) retinas. MRC2 expression in RMG was quantified as MFI of immunohistochemical staining (panel (**c**)). Retinal structure was visualized using DIC microscopy in both healthy (**A**) and diseased (**B**) retinas. Vimentin (green) labeled the structure and morphology of RMG in healthy (**C**) and diseased (**D**) retinas. In diseased retinas, RMG exhibited a more prominent gliotic phenotype, indicative of the early stage of an inflammatory episode (**D**). MRC2 (red) showed stronger expression in healthy retinas, particularly in RMG, but also along the OLM, OPL, IPL, and ILM (**E**). In contrast, MRC2 expression was almost absent in uveitic retinas (**F**). In healthy RMG, MRC2 expression was markedly stronger in the endfeet (marked with *), compared to the inner stem processes (marked with x). In uveitic RMG, MRC2 expression was restricted to the inner stem processes (x) and distal processes in the ONL. Overlay images (**G**,**H**) of Vimentin (green) and MRC2 (red) confirmed colocalization in RMG. Images were acquired at 630× magnification, and cell nuclei were counterstained with DAPI. Representative images were selected from biological replicates that best displayed the observed expression patterns. MRC2 expression was 3.7-fold higher in healthy RMG (panel (**c**), pink bar, left), compared to uveitic RMG (panel (**c**), red bar, right). The quantified difference in expression was statistically significant (* *p* = 0.0169). The asterisk indicates the level of statistical significance with 0.01 < * *p* ≤ 0.05. The bar plot displays the factor MFI value ± SD based on quantification of MFI in RMG from both controls and uveitis cases. Retinal layers are listed from innermost to outermost as follows: inner limiting membrane (ILM), ganglion cell layer (GCL), inner plexiform layer (IPL), inner nuclear layer (INL), outer plexiform layer (OPL), outer nuclear layer (ONL), outer limiting membrane (OLM), and photoreceptor outer segments (POS).

ARG1 was expressed in all healthy RMG, albeit with varying intensities (Figure 3E). In healthy RMG, the endfeet (Figure 2E, marked with *) exhibited moderate ARG1 expression, while expression intensity decreased in the inner stem processes (Figure 2E, marked with x). Expression levels increased again in the perikarya located in the INL and in the distal processes of RMG extending into the ONL (Figure 2E). In uveitic RMG, ARG1 expression was markedly stronger in all RMG (Figure 2F). While most uveitic RMG endfeet exhibited weak ARG1 expression (Figure 2F, marked with *), it was nearly absent in some RMG endfeet (Figure 2F). ARG1 expression increased consistently in the inner stem processes (Figure 2F, marked with x) and perikarya in the INL (Figure 2F). In the ONL, where RMG distal processes are located, ARG1 expression displayed a spotted and irregular pattern (Figure 2F). Overlay images of Vimentin and ARG1 confirmed ARG1 colocalization with RMG in both healthy (Figure 2G) and uveitic retinas (Figure 2H). The altered spatial distribution patterns and elevated expression in uveitic retinas underscore substantial changes in ARG1 expression during retinal neuroinflammation.

ARG1 expression in RMG of healthy horses and ERU cases was quantified by measuring the MFI of immunohistochemical staining. Quantification of the ARG1 expression revealed a 3.3-fold higher expression in uveitic RMG (panel c, red bar, Figure 2) compared to healthy controls (panel c, pink bar, Figure 2). This difference in expression was statistically significant (* *p* = 0.0351).

### 3.3. Spatial Distribution and Expression of MRC2 in Equine RMG with Significantly Higher Expression in Healthy RMG

We investigated the spatial distribution and expression of MRC2 in healthy retinas (Figure 3A, DIC) and retinas from uveitis cases (Figure 3B, DIC). To the best of our knowledge, MRC2 expression has not been previously reported in the retina of any species. The intermediate filament marker vimentin was used to visualize the localization and morphology of RMG (Figure 3C,D). This study focused on examining RMG during the early stages of inflammatory episodes.

In healthy retinas, RMG exhibited their characteristic columnar shape (Figure 3C). In uveitic retinas, RMG retained their columnar morphology but appeared more prominent, consistent with mild gliosis indicative of the early phase of an inflammatory response (Figure 3D). In healthy retinas, MRC2 was strongly expressed in multiple retinal layers and in RMG (Figure 3E). Distinct expression was observed along the OLM and outer plexiform layer (OPL), with even expression detected in the inner plexiform layer (IPL) and ILM (Figure 3E). MRC2 was also strongly expressed in photoreceptor outer segments (POS). In contrast, MRC2 expression was largely absent in most layers of the uveitic retina. Residual expression was confined to certain areas of the ONL and partially to RMG (Figure 3F).

MRC2 expression in healthy RMG was prominent in the endfeet (Figure 3E, marked with *), but slightly reduced in the inner stem processes (Figure 3E, marked with x), and in the cell perikarya within the INL (Figure 3E). Distal processes in healthy RMG exhibited renewed increased MRC2 expression (Figure 3E). In uveitic RMG, however, MRC2 expression showed a marked reduction and altered spatial distribution (Figure 3F). Most RMG displayed MRC2 expression limited to the inner stem processes (Figure 3F, marked with x), with some RMG showing little to no discernible expression (Figure 3F). The observed reduction and altered distribution of MRC2 in uveitic RMG highlight a significant contrast to the healthy retina, emphasizing the impact of inflammation on MRC2 expression.

To quantify the altered expression of MRC2 in equine RMG, we measured the expression of MRC2 as MFI of immunohistochemical staining in both healthy and diseased specimens. MRC2 expression was 3.7-fold higher in healthy RMG (Figure 3, panel c, pink bar) compared to uveitic RMG (Figure 3, panel c, red bar). The difference in expression was statistically significant (* *p* = 0.0169).

## 4. Discussion

This study sheds new light on the immune functions of RMG and their role in the pathogenesis of autoimmune uveitis. Using a spontaneous equine model of recurrent autoimmune uveitis, our discovery proteomics approach revealed distinct expression profiles between healthy and uveitic RMG, enabling a distinction between non-activated and activated states that have an inflammatory phenotype. Moreover, we identified several immune-related proteins in RMG. Alongside proteins with known associations to antigen presentation like MHC class II, we discovered previously unreported proteins with potential roles in retinal immunity and antigen presentation, such as MRC2. These findings highlight the complex and potentially immunomodulating role of RMG in the ocular immune response, expanding our understanding of the molecular mechanisms driving inflammation and the breakdown of ocular immune privilege in autoimmune uveitis. Our study focused on the expression of ARG1 and MRC2 in the initial stage of disease, characterized by only mild gliotic changes. Further functional investigations are needed to elucidate their role in ERU pathogenesis and to determine whether the expression levels of these markers correlate with disease progression and severity in later stages. The increased abundance of MHC class II in RMG from uveitis cases is a key indicator of RMG activation in autoimmune uveitis, highlighting the robustness of our approach (Table 1, Figure 1). Moreover, it strongly supports the hypothesis that these cells may act as atypical APCs in the retina. MHC class II is typically expressed on professional APCs such as macrophages, dendritic cells, and B cells, with its primary function being the presentation of antigens to CD4^+^ T cells [65,66]. CD4^+^ T cells are central players in the pathogenesis of autoimmune diseases [67]. In the context of autoimmune uveitis, the nature of the APC responsible for initiating and sustaining the CD4^+^ T cell response is still a subject of debate [46]. Although an eye–spleen axis has been discussed, suggesting the migration of eye-derived APCs to the spleen, thereby contributing to the activation of T cells against intraocular antigens in the periphery [68,69,70], the exact mechanisms behind this process remain unclear. Our findings corroborate earlier studies in horses with ERU, where MHC class II was localized to glial scars in the uveitic retina [41], which also aligns with similar results observed in subretinal fibrosis and uveitis syndrome in humans [52]. The hypothesis of RMG contributing to retinal inflammation via antigen presentation is further supported by recent findings of upregulated MHC class II in RMG in murine models of EAU, following immunization with the retinal autoantigen interphotoreceptor retinoid-binding protein [50,51]. Apart from MHC class II, uveitic RMG exhibited upregulation of various chemokines and leukocyte adhesion molecules, suggesting RMG to be the primary interactors with infiltrating leukocytes in the uveitic retina [50].

To fully activate CD4^+^ T cells, professional APCs express a variety of co-stimulatory and adhesion molecules alongside MHC class II [65]. In this study, we detected that equine RMG also constitutively expressed several costimulatory and adhesion molecules essential for the activation and adhesion of CD4^+^ T cells, such as CD40, ICAM1, CD81, CD9, CD48, and CD58 [65,71,72,73,74] (Figure S1). Since autoreactive uveitic CD4^+^ T cells are known to be activated in the periphery [34], these already activated cells may rely less on co-stimulation for reactivation, as observed in other autoimmune diseases such as multiple sclerosis and primary biliary cirrhosis [75,76,77,78,79]. Moreover, atypical APCs may employ different mechanisms for co-stimulation altogether: For instance, neutrophilic granulocytes, which can also act as atypical APCs under specific circumstances [80], have been shown to facilitate T cell co-stimulation via the CD58–C2-axis as an alternative to the classical co-stimulatory B7.1/B7.2-CD28 pathway observed in professional APCs [81]. Notably, RMGs also constitutively express CD58 (Figure S1), suggesting that a similar alternative co-stimulatory mechanism may operate in the retina [81]. Furthermore, the constitutive expression of lysosomal molecules LAMP1 and LAMP2 in equine RMG shows a potential for antigen processing by RMG (Figure S1) [82,83]. Moreover, the expression of pro-inflammatory cytokines is a critical step in CD4^+^ T cell activation [84]. Uveitic RMG express various interferon-induced and -related proteins (Table 1), as well as IFN-γ [16]. However, proof of functional antigen presentation by RMG in vivo is difficult to obtain [50]. An important question for future studies is to investigate how RMGs present antigens via the described receptors and whether classical or atypical co-stimulatory mechanisms are involved in the reactivation of autoreactive CD4+ T cells in the retina. These observations underscore the potential role of RMG in initiating and sustaining retinal inflammation through antigen presentation as well as the importance of the specific autoantigens involved in this process. Given that RMGs are strategically positioned to interact with infiltrating immune cells during retinal inflammation, further studies should explore the precise mechanisms by which MHC class II expression is upregulated in these cells during uveitis. This could provide valuable insights into the inflammatory processes that lead to the breakdown of immune privilege in the inner eye.

Furthermore, ARG1 emerges as another protein linked to activated RMG, with its higher expression in RMG from ERU cases (Figure 1 and Figure 2, Table 1). This is a novel discovery in an autoimmune uveitis model with spontaneous onset. ARG1 is an enzyme of the hepatic urea cycle, which plays a pivotal role in regulating inflammation by modulating nitric oxide (NO) production [85]. Furthermore, ARG1 is a key polarization marker for anti-inflammatory M2 macrophages in mice [61,86]. The specific function of ARG1, however, depends strongly on cell type, tissue and species [85]. In the context of autoimmune uveitis, a higher abundance of ARG1 has been associated with infiltrating myeloid cells, especially macrophages, in the uveitic retina of rodents with EAU [87,88]. An increased expression of ARG1 in RMG similar to our findings in ERU was not detected in these EAU models [87,88]. In other uveitis models, however, increased levels of ARG1 in RMG was evident [89]. Notably, in mouse models of endotoxin induced uveitis and diabetic retinopathy, an elevated expression of ARG1 in RMG has been associated with immune regulated pro-inflammatory responses such as the uncoupling of enzymes of the nitric oxide synthase (NOS)-family [89,90]. This uncoupling was proposed to arise from substrate competition between isoforms of NOS (inducible and endothelial) and ARG1 for their shared substrate L-arginine, leading to an increased production of ROS and reactive nitrogen species, potentially exacerbating retinal pathology [89,90]. Such imbalances between ARG1 and NOS are context- and tissue-dependent but often result in tissue damage [91]. Notably, no isoforms of the NOS family were detected in the equine RMG proteome, indicating that enzymatic activity in uveitic RMG may shift to ARG1 in this context. This shift likely reflects a metabolic and immunologic adaptation where ARG1 takes on a central role in modulating inflammation and oxidative stress. Without NOS activity, L-arginine metabolism dominated by ARG1 could lead to ROS production through the oxidation of ARG1′s downstream metabolites, like polyamines [92,93]. Moreover, these polyamines, which are produced downstream of L-ornithine metabolism [85], may further influence the inflammatory processes in the uveitic retina, as they have been implicated in neural excitotoxicity and immune cell function, particularly in T cells [94,95,96]. Given the neural excitotoxicity of polyamines downstream of ARG1 in specific contexts, such as in the rodent retina following intraocular injection of *N*-methyl-D-aspartate or during retinal ganglion cell exposure to polyamine degradation products [96,97,98], the question has to be raised if polyamines potentially also affect retinal neurons through mechanisms like excitotoxicity in the course of uveitis pathogenesis. Furthermore, polyamines could play a role in modulating the proliferation and differentiation of CD4^+^ T cells, especially the Th17 subset, which has been linked to autoimmune diseases and their respective rodent models, including autoimmune uveitis in humans and EAU in mice [99,100], and has also been discussed to contribute to ERU pathogenesis [101]. The high abundance of ARG1 in RMG may therefore play a crucial role in shaping the inflammatory response in ERU. Its immunomodulatory effects, particularly on CD4^+^ T cell biology and RMG–neuron interaction, should be investigated in more detail in future studies.

Since ARG1 is expressed by professional APCs, with a regulatory role in immune response and inflammation [85], this protein may similarly play a key role in regulating immune responses in the retina, especially under inflammatory conditions. Measuring levels of NO and ROS—downstream metabolites of ARG1 and NOS—in vitreal samples could provide valuable insights into inflammatory processes mediated by ARG1 activity. Together with MHC class II, ARG1 is a marker for RMG activation and the inflammatory and potentially antigen-presenting phenotype. Moreover, it could be a promising target for modulating retinal inflammation in autoimmune uveitis.

RMGs play a dual role as pivotal drivers of inflammation and vital regulators of structural integrity and immune balance in the retina. Identifying distinct markers that differentiate activated RMG from their healthy counterparts is essential for accurately distinguishing retinal health from disease. Our identification of matricellular receptor MRC2 (also known as uPARAP/Endo180/CD280) (Figure 1 and Figure 3, Table 2 is an entirely novel finding in RMG, since, to the best of our knowledge, MRC2 has not been previously identified in RMG of any species. Consequently, its role in retinal health and pathologies remains unexplored to date. MRC2 has been described as a regulator of vascular endothelial growth factor (VEGF) receptor functionality in a murine model of pathological lymphangiogenesis by restricting VEGF receptor heterodimerization, a process essential for both lymphatic and vascular endothelial growth [102,103]. Decreased abundance of MRC2 in ERU and re-distribution away from the RMG endfeet (Figure 3) may therefore impact retinal vascular remodeling and contribute to the breakdown of retinal immune privilege during retinal neuroinflammation, potentially facilitating disease progression. Moreover, the robust and high expression of MRC2 in RMG from healthy retinas shows the potential of this protein as a prospective biomarker for RMG health.

Along with MRC2, we identified THBS1 as a protein significantly more abundant in healthy RMG (Figure 1, Table 2). THBS1, an anti-angiogenic factor, is known to regulate extracellular matrix (ECM) homeostasis [104] and suppresses retinal neovascularization in models of diabetic retinopathy [105,106,107,108]. Its significantly lower abundance in uveitic RMG supports the idea that ECM regulation is disrupted in ERU [109]. Given its anti-angiogenic properties, the downregulation of THBS1 in ERU may exacerbate retinal vascular leakage and neovascularization, which are hallmarks of ERU pathogenesis [110,111], thereby contributing to the breakdown of the ocular immune privilege. Among its pleiotropic properties, THBS1 also exerts immunosuppressive functions under certain conditions. In murine EAU, topically applied THBS1-derived peptide significantly reduced uveitis pathology and retinal leukocyte adhesion [112]. THBS1 is also a potent activator of Transforming Growth Factor β (TGFβ). In a murine EAU model, THBS1-mediated activation of TGFβ contributed to the amelioration of retinal inflammation [113]. Additionally, the knockout of THBS1 in a murine model of dry eye disease (Sjögren’s syndrome) led to elevated levels of Th17 cells in the lacrimal gland, exacerbating inflammation [114]. THBS1 in RMG may thus act as a key mediator of immunosuppression in the retina during ERU. By regulating T cell infiltration and promoting an immunosuppressive environment, THBS1 likely plays a critical role in modulating the retinal immune response. Interestingly, MRC2 has been shown to facilitate the endocytosis of THBS1 in murine fibroblasts [64], suggesting a potential interplay between MRC2 and THBS1 that may be crucial for ECM regulation, the preservation of the ocular immune privilege, and an immunosuppressive environment.

MRC2 has also been identified as a marker for M2 macrophages and plays a role in the uptake and clearance of ECM components like collagens [115,116,117]. Additionally, it is also involved in the clearance of collectins (C-type lectins), like Mannose Binding Lectin (MBL) and Collectin 11 in murine fibroblasts, implicating MRC2 as an immunoregulatory protein in these cells [118,119]. Collectins are soluble pattern recognition molecules in the innate immune system [120,121,122]. MBL, for instance, can function directly as an opsonin by binding to pathogens independently of complement activation, thus enabling opsonophagocytosis by human macrophages [123,124]. This could aid in processing antigens for presentation to the adaptive immune system in APCs. Moreover, circulating MBL has been described to be essential for antigen presentation by human dendritic cells, as shown in patients with MBL deficiency, where dendritic cells exhibited a diminished capacity to induce T cell responses [125]. Besides this, collectins are also potent activators of the complement system [122]. Interestingly, complement system activation has been described to play a key role in autoimmune uveitis in humans [126] and is also discussed to contribute to the pathogenesis of ERU [111,127]. Assuming a similar role of MRC2 in RMG as described in murine fibroblasts [119], a reduced abundance of MRC2 in ERU may impair the clearance of collectins as pattern recognition molecules, potentially exacerbating retinal inflammation, whereas high expression of MRC2 in healthy RMG may suppress complement system activation, thus protecting and sustaining retinal health.

Additionally, the high MRC2 levels in healthy RMG might contribute to maintaining an immunosuppressive retinal environment, as previously shown in murine cancer-associated fibroblasts expressing high amounts of MRC2 [128]. These fibroblasts were attributed with immunosuppressive properties by preventing CD8^+^ T cell infiltration, thereby rendering tumors immunologically “cold” [128]. Correspondingly, lower MRC2 expression in ERU could disrupt this immunosuppressive function, promoting T cell infiltration. In a murine model of endometriosis, the high abundance of MRC2 in endometrial stromal cells was found to be essential for the differentiation of regulatory T cells (Tregs) in coincubation experiments [129]. Tregs, a specialized subset of CD4^+^ T cells, play a crucial role in maintaining immune homeostasis and have been shown to ameliorate EAU in mice [130]. Their involvement in autoimmune uveitis in humans has also been reported, with patients exhibiting decreased levels of peripheral Tregs during acute inflammation, compared to phases of quiescence [131,132]. Since a similar role of Tregs has been discussed in the pathogenesis of ERU [34], the involvement of MRC2 in Treg differentiation and, consequently, the preservation of an immunosuppressive environment in the healthy retina warrants further investigation.

Regarding their function in other cells and tissues, the identification of MRC2 and its interactor THBS1, both with high expression in healthy RMG, suggests these proteins as promising novel candidate markers for immune balance and functional integrity in the retina. Specifically, the novel identification of a higher abundance of MRC2 in RMG could imply a potential contribution to ocular immune privilege by regulating vascular and collectin homeostasis. The reduced abundance of MRC2 in uveitic RMG reflects a shift in their immunological and functional profile during autoimmune uveitis.

C-type lectin scavenger receptors are involved in antigen capture and the endocytosis of glycoproteins, which can subsequently be processed and cross-presented via MHC class I [133,134]. Although MRC2, a member of the C-type lectin receptor family, has not yet been specifically investigated for its role in this process, it could potentially contribute to antigen cross-presentation via MHC class I in healthy RMG. Notably, MHCB3, an MHC class I heavy chain, is differentially expressed in healthy RMG. Previous bioinformatic analyses of cancer-associated fibroblasts have highlighted MRC2 as a potential biomarker for fibroblasts with antigen-presenting capabilities [135]. Although the involvement of MRC2 in antigen presentation processes remains context-dependent, its association with this function underscores the need for further research into its role in immune-related processes such as antigen cross-presentation, particularly in potential atypical APCs like RMG Recent research on murine macrophages has demonstrated that rather than conforming to strict polarization subtypes, macrophage activation occurs within a certain spectrum, comprising characteristics of both M1 and M2 phenotypes in varying nuances [62,136]. This underscores the plasticity of APCs. A similar principle may apply to RMG, which, as shown in our study, express markers commonly associated with professional APCs such as MHC class II and ARG1. These molecules significantly decrease in RMG from healthy retinas, while MRC2 is highly abundant in healthy RMG and decreases in uveitis. These findings indicate that APC characteristics may be governed by different molecules depending on RMG phenotype and surroundings. This potential dichotomy in RMG suggests that in a healthy state, RMG may predominantly present antigen via MHC class I potentially aided by the endocytic receptor MRC2. In contrast, RMGs from uveitic retina shift antigen presentation towards MHCII and ARG1. However, further research is necessary to fully characterize the functional implications of this potential plasticity in RMG and their role in retinal autoimmune responses.

To fully assess the role of ARG1 and MRC2 for disease pathogenesis, future studies are needed involving in vivo knockdown or overexpression approaches, which are technically feasible in rodent models. Despite translational limitations of rodent models [37,137], such experiments would greatly enhance our understanding of the molecular mechanisms involving RMG in autoimmune uveitis. This study provides the foundational work for further functional investigations on the role of these proteins in RMG in autoimmune uveitis.

Despite the comprehensive analysis provided by differential proteomics and immunohistochemistry, each method has certain limitations. Label-free quantitative proteomic methods, despite recent technical advances, still face challenges in achieving accurate and high-quality quantification, especially compared to label-based approaches [138,139]. This is primarily due to the asymmetrical distribution of protein abundances, where high-abundance proteins dominate the analysis, and the presence of missing values, which arise from biological, technical, or analytical factors [139,140]. Moreover, low-abundance proteins that fall below the detection and quantification limits are often excluded from the analysis [141,142]. Immunohistochemistry, while effective for localizing proteins, is limited by issues related to antibody specificity, potential artifacts, and its semi-quantitative nature. To overcome these challenges, it is essential to combine multiple methods, refine analytical techniques, and apply robust data integration strategies to obtain more accurate and comprehensive insights into protein dynamics. Therefore, a combination of label-free differential proteomics and immunohistochemistry, as applied in this study, provides complementary insights by leveraging the strengths of both techniques for enhanced target protein analysis.

## 5. Conclusions

In conclusion, this study highlights the crucial role of RMG in autoimmune uveitis, particularly in ERU. Our findings demonstrate that activated RMG express key immune-related proteins, such as MHC class II and ARG1, suggesting their involvement in antigen presentation and inflammation modulation. The identification of MRC2, a receptor implicated in both immune regulation and potentially antigen presentation, introduces a novel aspect of the immunological functions of RMG. MRC2’s role in modulating the immune response, coupled with its reduced expression in uveitic RMG, points to immune dysregulation in the retina. Additionally, THBS1, known for its immunosuppressive properties, is significantly reduced in ERU, further emphasizing the breakdown of immune regulation in retinal inflammation. These proteins, especially MRC2 and THBS1, emerge as potential markers for assessing RMG activation, retinal immune balance, and the maintenance of ocular immune privilege in autoimmune uveitis. Overall, these findings provide deeper insights into the molecular mechanisms of retinal inflammation and suggest potential therapeutic strategies aimed at modulating RMG function to restore immune homeostasis and ocular health in autoimmune uveitis.

## Figures and Tables

**Table 1:** Proteins of interest with association to the immune system that showed a higher abundance (≥2) in uveitic RMG compared to healthy controls. Proteins that were selected for further analysis are highlighted in bold letters. *p*-values were adjusted with the Benjamini–Hochberg correction to account for multiple comparisons (adj. *p*). Column 1 lists the protein name, while column 2 provides the corresponding gene names, Column 3 (Accession Number) shows the protein accession numbers from the Ensembl horse database (Version 75), Column 4 (Adj. *p*-value) displays the *p*-values adjusted for multiple testing. Column 5 (Ratio ERU/healthy) shows the ratio of protein abundance in ERU RMG compared to healthy control cells.

Protein	Gene Name	Accession Number	Adj. *p*-Value	Ratio ERU/Healthy
Regulatory Factor X5	RFX5	ENSECAP00000022315	7.4294 × 10^−17^	100
Serum Amyloid A1	SAA	ENSECAP00000009324	7.4294 × 10^−17^	100
Small Ubiquitin-like Modifier 1	SUMO1	ENSECAP00000022200	7.4294 × 10^−17^	100
Major Histocompatibilty complex class II antigen DR beta chain	MHC II DRB	ENSECAP00000019909	7.4294 × 10^−17^	100
Major Histocompatibilty complex class II antigen DR alpha chain	MHC II DRA	ENSECAP00000010541	7.4294 × 10^−17^	100
Toll-like Receptor 3	TLR3	ENSECAP00000000146	7.4294 × 10^−17^	100
Recoverin	RCVRN	ENSECAP00000016469	7.4294 × 10^−17^	100
S-antigen	SAG	ENSECAP00000012776	7.4294 × 10^−17^	100
Interferon Induced Protein 1	IFIT1	ENSECAP00000003048	7.4294 × 10^−17^	46
Ly1 Antibody Reactive	LYAR	ENSECAP00000010643	7.4294 × 10^−17^	29.6
Interferon-induced Protein 44-like	IFI44L	ENSECAP00000007567	1.1967 × 10^−10^	13.6
Interferon-induced protein 44	IFI44	ENSECAP00000006938	1.1796 × 10^−9^	12.2
S100 calcium binding protein A7	S100A7	ENSECAP00000006669	2.023 × 10^−5^	8.7
Lamin A/C	LMNA	ENSECAP00000009322	5.607 × 10^−6^	8.2
Interferon induced with Helicase C Domain 1	IFIH1	ENSECAP00000006405	5.5493 × 10^−7^	5.7
ISG15 Ubiquitin-like Modifier	ISG15	ENSECAP00000000924	1.2196 × 10^−6^	5.5
Arginase 1	ARG1	ENSECAP00000021081	0.0001	4.9
Neutrophil Cytosolic Factor 1	NCF1	ENSECAP00000012958	0.0201	3.2
Interferon-induced protein 4	IFIT4	ENSECAP00000008012	0.0129	3.2
Proteasome Subunit, Beta Type, 10	PSMB10	ENSECAP00000010204	0.0081	3
CXC Chemokine Motif Ligand 11	CXCL11	ENSECAP00000010001	0.0293	3
Interleukin Enhancer Binding Factor 2	ILF2	ENSECAP00000006934	0.0356	2.5

**Table 2:** Proteins of interest with an association to the immune system that showed a higher abundance (≥2) in control RMG compared to uveitic RMG. Proteins that were selected for further analysis are highlighted in bold letters. *p*-values were adjusted with the Benjamini–Hochberg correction to account for multiple comparisons (adj. *p*). Column 1 lists the protein name, while column 2 provides the corresponding gene names. Column 3 (Accession number) includes the accession numbers of the identified proteins from the Ensembl horse database (Version 75). Column 4 (Adj. *p*-value) displays the *p*-values, adjusted for multiple testing. Column 5 (Ratio healthy/ERU) shows the ratio of the protein abundance of control cells compared to ERU RMG.

Protein	Gene Name	Accession Number	Adj. *p*-Value	Ratio Healthy/ERU
Matrix Metallopeptidase 19	MMP19	ENSECAP00000008915	7.4294 × 10^−17^	100
Integrin, alpha 2	ITGA2	ENSECAP00000022803	1.4549 × 10^−6^	5.2
Mannose Receptor C-type 2	MRC2	ENSECAP00000010799	0.0001	4
Major Histocompatibility complex class I heavy chain	MHCB3	ENSECAP00000019780	0.0026	3.8
Thrombospondin 1	THBS1	ENSECAP00000007423	0.0148	3.6
Interleukin 1 Receptor Accessory Protein	IL1RAP	ENSECAP00000004063	0.0478	3
Integrin, beta 5	ITGB5	ENSECAP00000006461	0.0113	2.8
CD63 molecule	CD63	ENSECAP00000017837	0.0119	2.7
Integrin, alpha V	ITGAV	ENSECAP00000020901	0.0201	2.5
Leukocyte Elastase Inhibitor	SERPINB1	ENSECAP00000008115	0.0161	2.5
Cathepsin F	CTSF	ENSECAP00000019741	0.0353	2.4
Catalase	CAT	ENSECAP00000017490	0.0292	2.4

## Data Availability

The proteomic data are deposited in the PRIDE repository with the dataset identifier PXD058170. Reviewers can access the dataset at https://www.ebi.ac.uk/pride/ (accessed on 22 November 2024) using the following account details: Username: reviewer_pxd058170@ebi.ac.uk. Password: ifmpX647rjiv.

## Supplementary Materials

The following supporting information can be downloaded at: www.mdpi.com/article/10.3390/biom15020288/s1; Table S1: Quantified protein identifications from the equine RMG proteome by differential proteome analysis ERU vs. healthy controls; Table S2: Pathway analysis of differentially expressed proteins; Figure S1: MA-plot of non-differentially expressed proteins related to co-stimulation and antigen processing.
