# Supplementary material for: Differential Expression of ARG1 and MRC2 in Retinal Müller Glial Cells During Autoimmune Uveitis
Source: Biomolecules. 2025 Feb 14;15(2):288. doi: 10.3390/biom15020288 (PMC11853277; doi:10.3390/biom15020288)
Supplement: Supplementary file 1 [file biomolecules-15-00288-s001.zip › Figure S1_Fleischer_et_al_biomolecules3403378.pdf]

Supplementary Figure S1: MA-plot of non-differentially expressed proteins related to co-stimulation and antigen processing

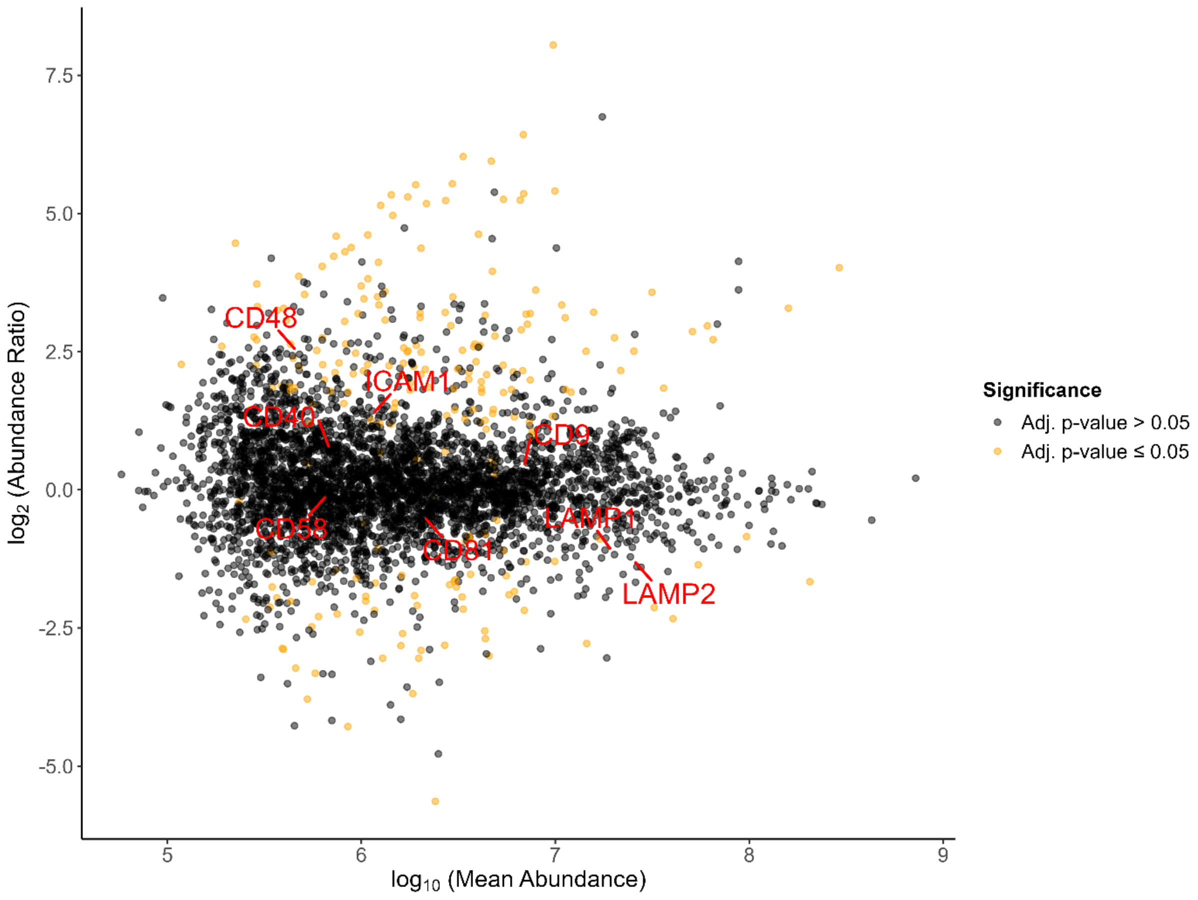

Figure S1: MA-plot visualizing the $\log_2$(Abundance Ratio) of proteins between ERU samples and control samples as a function of $\log_{10}$(Mean Abundance) across all measured samples. Each point represents a detected protein, with colors indicating statistical significance: proteins with an adjusted $p \leq 0.05$ are shown in orange, while non-significant proteins (adj. $p > 0.05$) are displayed in black. The co-stimulatory proteins and adhesion factors CD40, intracellular adhesion molecule 1 (ICAM1), CD81, CD9, CD48 and CD58, as well as the lysosome-associated membrane proteins (LAMP)1 and LAMP2 are specifically labeled in red.

The MA-Plot was generated with R (version 4.3.1, R Core Team (2024); Vienna, Austria, https://www.R-project.org) with the ggplot2 package (version 3.5.1).
